# Heterogeneity in the association between retirement and cognitive function: a machine learning analysis across 19 countries

**DOI:** 10.1093/ije/dyaf201

**Published:** 2025-11-24

**Authors:** Koryu Sato, Haruko Noguchi, Kosuke Inoue

**Affiliations:** Faculty of Policy Management, Keio University, Kanagawa, Japan; Graduate School of Economics, Waseda University, Tokyo, Japan; Department of Social Epidemiology, Graduate School of Medicine and School of Public Health, Kyoto University, Kyoto, Japan; Graduate School of Economics, Waseda University, Tokyo, Japan; Department of Health Promotion and Behavioral Sciences, Graduate School of Medicine and School of Public Health, Kyoto University, Kyoto, Japan

**Keywords:** retirement, state pension age, episodic memory, causal forests, instrumental variable

## Abstract

**Background:**

Rising state pension ages in many developed countries may influence cognitive aging by delaying retirement, yet the cognitive consequences of retirement likely vary across individuals and contexts. This study investigates the heterogeneous association between retirement and cognitive function.

**Methods:**

We analyzed harmonized data from three longitudinal studies: the Health and Retirement Study, the English Longitudinal Study on Ageing, and the Survey of Health, Ageing and Retirement in Europe. The dataset encompassed three waves across 19 counties from 2014 to 2019. Our study included 12 811 individuals who worked in the first wave, from whom each survey collected covariate information. We assessed retirement status among participants aged 50–80 years in the second wave and measured cognitive function using word recall tests in the third wave. The analysis employed instrumental variable causal forests estimation, utilizing state pension age as an instrument for retirement.

**Results:**

Among 7432 individuals with retirement propensity scores between 0.1 and 0.9, 2165 (29.1%) retired during the second wave. Analysis revealed that retirees recalled 1.348 more words than workers on average. The association between retirement and cognitive function was heterogeneous; greater cognitive benefits were observed among women, individuals with higher socioeconomic status, those with robust pre-retirement health, and those who engaged in physical activity before retirement.

**Conclusions:**

The observed heterogeneous associations suggest policymakers should consider incorporating early retirement options into the pension system, allowing individuals to make retirement decisions based on their circumstances.

Key Messages

- We employed instrumental variable causal forests to investigate the conditional average treatment effect of retirement on cognitive function.
- This study revealed previously unidentified heterogeneity in the retirement cognitive association dependent on individual characteristics and the countries studied, which suggests enhanced cognitive benefits among women, individuals with higher socioeconomic status, those with robust pre-retirement health, and individuals who engaged in physical activity before retirement.
- The heterogeneous nature of retirement’s effects suggests that policymakers should consider implementing early retirement provisions within pension systems, enabling retirement decisions based on individual circumstances.

## Introduction

The cognitive health of older adults is a growing global concern. Cognitive decline, particularly in episodic memory, often represents an early stage in the neurodegenerative process that, if progressive and accompanied by deficits in other cognitive domains, can eventually lead to dementia [1]. In 2015, approximately 47 million people were living with dementia, with projections indicating a 1.6-fold increase to 75 million by 2030 [2]. In response to rapidly aging populations, many developed countries are raising their state pension age (SPA). These policy changes may affect cognitive health by delaying retirement, thereby altering individuals’ budgetary constraints and time allocation between work and leisure in later life [3].

Despite numerous studies using identical datasets, the effects of retirement on cognition remain uncertain, with no clear consensus emerging [4, 5]. For example, studies using Health and Retirement Study (HRS) data have yielded contradictory findings, with some demonstrating a detrimental association of retirement with cognitive function [6, 7], whereas others show no association [8]. Similarly, research utilizing data from the English Longitudinal Study on Ageing (ELSA) or the Survey of Health, Ageing and Retirement in Europe (SHARE) has produced inconsistent results. While several studies reported harmful associations [9, 10], others found no relationship [11–14], and some even suggested beneficial associations [15, 16].

Several factors may contribute to these inconsistencies in the literature. First, model misspecification can influence effect estimates. A review replicating previous studies demonstrated that the choice of statistical methodologies is a key determinant of findings [5]. Additionally, effect heterogeneity introduces another source of variation. Studies have shown that the association between retirement and cognitive function varies by individual characteristics such as sex, education, and pre-retirement occupation [10, 12, 17–20]. When adverse effects in one subgroup mask positive effects in others, the overall population treatment effect becomes ambiguous. Nonetheless, the full extent of heterogeneity in this relationship remains poorly understood.

To address these challenges, we applied instrumental variable causal forests (IV forests) [21] to investigate the conditional average treatment effect (ATE) of retirement on cognitive function. We employed country-specific SPA as an instrument for retirement to address the endogenous nature of retirement decisions. The IV forests method incorporates random-forest-based nonparametric estimation, offering greater robustness against model misspecification compared to conventional parametric approaches. Our analysis utilized harmonized data from 19 countries and incorporated 60 covariates as potential confounders and effect modifiers. This machine learning-based approach enables us to uncover previously hidden effect heterogeneity in the association between retirement and cognitive function.

## Methods

### Study design and participants

This study used harmonized panel datasets from the HRS, ELSA, and SHARE provided by the Gateway to Global Aging Data project [22]. Our data encompassed three waves: covariates (excluding age) were obtained from the HRS and ELSA in 2014 and SHARE in 2015; age and labor force status were ascertained via the HRS and ELSA in 2016 and SHARE in 2017; and cognitive function outcomes were assessed in the HRS and ELSA in 2018 and SHARE in 2019. To prevent retirement from influencing covariates, we collected covariate information from the wave preceding retirement status measurement. Furthermore, we measured outcomes at least 2 years after retirement, acknowledging the potential temporal lag in retirement’s impact on cognitive function [12].

Among 94 824 individuals who participated in the first wave, 49 555 completed all three waves. We included 43 052 individuals aged 50–80 years from the second wave, but excluded 29 519 individuals who were not working in the first wave and 722 individuals who neither worked nor retired in the second wave (e.g. unemployed, disabled, or homemaker). The final sample comprised 12 811 participants for IV forests development (Supplementary Fig. S1). To assess the potential impact of attrition, we compared baseline characteristics between included and excluded individuals (12 033 individuals who aged under 78 years and worked in the first wave but were excluded from the analysis). Supplementary Table S1 shows that there was no difference in the baseline cognitive function between included retirees and excluded individuals.

### Cognitive function assessment

Episodic memory was assessed as a measure of cognitive function. Episodic memory involves the ability to recall past experiences, which typically declines with age [23]. The assessment followed the Consortium to Establish a Registry for Alzheimer’s Disease (CERAD) Battery protocol [24]. Participants listened to 10 common words and were immediately asked by an interviewer to recall as many words as possible. After approximately 5 minutes, they were asked to recall the words again. Hence, the total number of words recalled, ranging from 0 to 20, represented their episodic memory function, consistent with previous studies [5–8, 10–13, 15, 16].

### Retirement status classification

Labor force status was self-reported in the surveys (Supplementary Method S1). We focused on individuals who were working during the first wave. In the second wave, we defined retirees as those who self-identified as “retired” or “partly retired,” regardless of current working status, aligning with previous literature (Supplementary Table S2) [15, 17, 20, 25]. While some studies have defined retirement simply as not working [6, 10, 11, 14], we tested our findings’ robustness using this narrower definition.

### State pension age implementation

To address potential endogenous retirement decision bias, we employed the SPA as an IV for retirement (Supplementary Table S3). Following previous research [11, 20, 25, 26], we used joint instruments including the early retirement age (ERA) and official retirement age (ORA). The binary ERA variable indicated whether participants had reached the earliest age for receiving reduced or full pension benefits under specific conditions. The ORA variable denoted whether participants had reached the age for receiving a minimum guaranteed or full pension without conditions. For countries without early retirement schemes, the ERA variable was set to zero.

The SPA serves as a valid IV by increasing retirement probability (relevance condition) without directly affecting cognition (exclusion restriction condition). Assuming monotonicity, the point estimate represents a local ATE among individuals who would retire upon reaching SPA. Our study leverages within-country SPA variations (institutional differences across birth cohorts and sexes) and between-country variations using harmonized data. Retirement rates demonstrably increase around the SPA (Supplementary Figs S2 and S3), supporting the relevance condition.

### Statistical analysis

We compared ATEs using parametric ordinary least squares (OLS), two-stage least squares (2SLS), nonparametric causal forests without IVs (non-IV forests), and IV forests. For parametric methods, we adjusted for 10 covariates selected by importance in trained IV forests. ATEs for nonparametric methods were obtained through residual-on-residual regressions [27].

The IV forests method combines the generalized method of moments for IV estimation with random forests to identify similar treatment effects (Supplementary Method S2). The IV forests incorporated 60 harmonized covariates from the first wave (Supplementary Tables S4 and S5). To mitigate potential reverse causality, we included the baseline score of cognitive function. Missing values were imputed using a random forests-based algorithm [28], assuming data missing at random (Supplementary Table S6). After training, we restricted the analysis to 7432 individuals with retirement propensity scores between 0.1 and 0.9 [29], as extreme scores destabilize estimation. Our estimand of the IV forests represents the conditional local ATE on the overlap population (CLATO).

We then categorized observations into quintiles from Q1 (the lowest CLATO; least retirement benefits) to Q5 (highest CLATO; most retirement benefits), and compared sociodemographic characteristics, health and behaviors, and countries across quintiles. *P*-values were adjusted using the Bonferroni method. All analyses were performed using R 4.1.3 (R Foundation for Statistical Computing, Vienna, Austria).

### Sensitivity analyses

We performed several sensitivity analyses to confirm the robustness of our findings. These included: restricting the sample to ages 55–75, excluding partially retired individuals, analyzing only full-time employees, and excluding US data with the largest number of samples.

## Results

### Descriptive statistics

The study population comprised 7432 individuals, of whom 2165 (29.1%) were retirees in the second wave (Table 1). On average, workers could recall 11.1 words, while retirees could recall 10.8 words in the third wave. The outcome showed a normal distribution (Supplementary Fig. S4).

**Table 1.** Descriptive statistics of the overlap population

Variables, *n* (%)	Worker *n* = 5267 (70.9%)	Retiree *n* = 2165 (29.1%)
Sociodemographic characteristics		
Age (years), mean (SD)	63.9 (3.86)	65.8 (4.27)
Men	2667 (50.6)	1063 (49.1)
Foreign-born	707 (13.4)	230 (10.6)
Education, mean (SD)	2.2 (0.68)	2.1 (0.69)
Married	4059 (77.1)	1663 (76.8)
Living alone	934 (17.7)	406 (18.8)
No children	544 (10.3)	200 (9.2)
≥3 children	1885 (35.8)	760 (35.1)
Asset, z-score, mean (SD)	0.1 (1.21)	0.0 (0.79)
Income, z-score, mean (SD)	0.1 (1.14)	0.0 (0.91)
Professional	2272 (43.1)	878 (40.6)
Clerk	793 (15.1)	329 (15.2)
Service and sales	1067 (20.3)	446 (20.6)
Manual labor	1140 (21.6)	515 (23.8)
Physical demand, mean (SD)	2.3 (1.07)	2.3 (1.05)
Part-time job	1298 (24.6)	775 (35.8)
Self-employed	1151 (21.9)	368 (17.0)
Health and behaviors		
Baseline cognition, mean (SD)	11.3 (3.19)	11.0 (3.26)
Self-rated health, mean (SD)	3.4 (0.98)	3.3 (0.95)
Depression, z-score, mean (SD)	0.0 (1.01)	0.0 (0.97)
Life satisfaction, z-score, mean (SD)	0.0 (1.00)	0.1 (0.98)
Hypertension	2064 (39.2)	982 (45.4)
Diabetes	626 (11.9)	311 (14.4)
Cancer	372 (7.1)	177 (8.2)
Lung disease	231 (4.4)	122 (5.6)
Heart disease	571 (10.8)	265 (12.2)
Stroke	107 (2.0)	69 (3.2)
Arthritis	1633 (31.0)	800 (37.0)
Psychiatric problems	542 (10.3)	244 (11.3)
Hyperlipemia	1534 (29.1)	634 (29.3)
Health limitations in working	526 (10.0)	291 (13.4)
Difficulty in ADL	205 (3.9)	137 (6.3)
Difficulty in IADL	129 (2.4)	45 (2.1)
Distance eyesight, mean (SD)	3.8 (0.94)	3.8 (0.92)
Near eyesight, mean (SD)	3.6 (0.98)	3.6 (0.97)
Hearing, mean (SD)	3.6 (1.00)	3.5 (0.99)
Pain problems	1702 (32.3)	783 (36.2)
Obesity	1515 (28.8)	612 (28.3)
Physical activity	4629 (87.9)	1888 (87.2)
Heavy drinking	524 (9.9)	237 (10.9)
Smoking	795 (15.1)	369 (17.0)
Countries		
Austria	50 (0.9)	37 (1.7)
Belgium	99 (1.9)	58 (2.7)
Croatia	37 (0.7)	14 (0.6)
Czech Republic	147 (2.8)	126 (5.8)
Denmark	291 (5.5)	92 (4.2)
Estonia	379 (7.2)	93 (4.3)
France	123 (2.3)	86 (4.0)
Germany	262 (5.0)	128 (5.9)
Greece	182 (3.5)	49 (2.3)
Israel	100 (1.9)	30 (1.4)
Italy	121 (2.3)	37 (1.7)
Luxembourg	44 (0.8)	27 (1.2)
Poland	31 (0.6)	13 (0.6)
Slovenia	94 (1.8)	60 (2.8)
Spain	108 (2.1)	61 (2.8)
Sweden	263 (5.0)	152 (7.0)
Switzerland	276 (5.2)	115 (5.3)
England	974 (18.5)	403 (18.6)
United States	1686 (32.0)	584 (27.0)
Outcome		
Cognitive function, mean (SD)	11.1 (3.21)	10.8 (3.19)

ADL, activities of daily living; IADL, instrumental activities of daily living. Imputed data are used.

### Association between retirement and cognitive function

Initial analyses using OLS and non-IV forests revealed no evidence of the association between retirement and cognitive function (Table 2). However, 2SLS presented a positive association between retirement and word recall (0.962, 95% confidence interval [CI]: 0.287–1.637). The validity of our IVs was supported by an F statistic of 163.037 (*P *< .0001) and a Sargan statistic of 1.177 (*P *= .28). IV forests corroborated these findings, indicating that retirees recalled 1.348 more words than workers (95% CI: 0.313–2.384). Variable importance metrics from the IV forests are presented in the Supplementary Fig. S5.

**Table 2.** Average treatment effects of retirement on cognitive function

	(1) OLS^a^	(2) 2SLS^a^	(3) Non-IV forests	(4) IV forests
Retirement	−0.013	0.962	−0.031	1.348
95% CI	(−0.150 to 0.123)	(0.287 to 1.637)	(−0.171 to 0.108)	(0.313 to 2.384)
Observations	7432	7432	7432	7432
F statistic		163.037		
Sargan statistic		1.177		

a The model is adjusted for the following covariates selected based on variable importance in trained IV forests: assets, age, income, baseline cognition, depression, life satisfaction, self-rated health, hearing, degree of the job’s physical demands, and distance eyesight.

### Heterogeneity across individual characteristics and countries

The distribution of conditional ATEs, detailed in the Supplementary Fig. S6, showed marked differences between non-IV and IV forests. While non-IV forest estimates clustered around zero, IV forest estimates exhibited heterogeneous distribution. The ATEs increased monotonically with CLATO ranking from Q1 to Q5, suggesting that IV forests captured heterogeneity in retirement’s effect on cognitive function (Supplementary Fig. S7).

**Figure 1.**
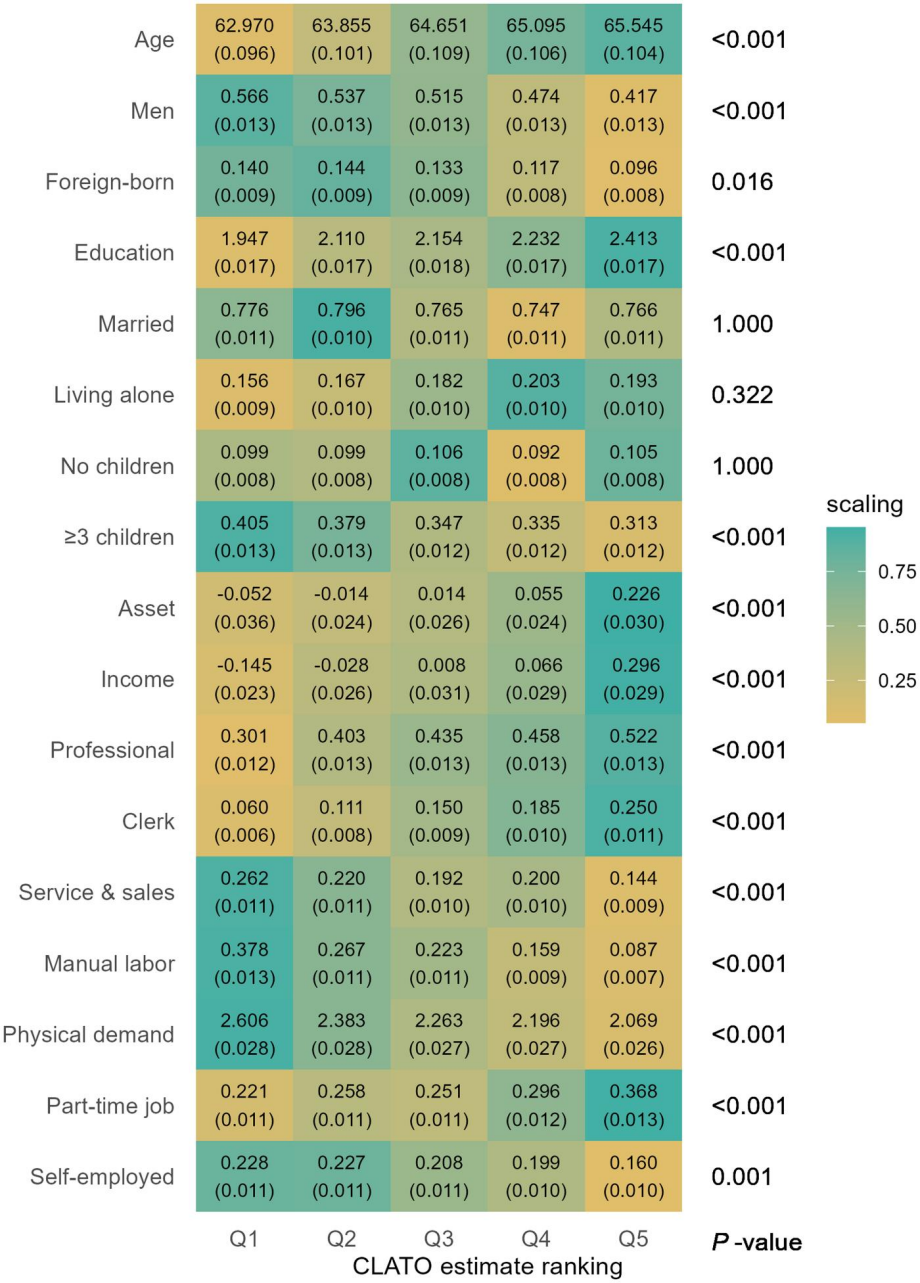
Heterogeneity in sociodemographic characteristics. Q1 represents the group with the lowest conditional local average treatment effect on the overlap population (CLATO), whereas Q5 represents the group with the highest CLATO. Each tile indicates the mean value of a covariate within the group, with its standard deviation in parentheses. “Scaling” assigns 1 (green) to the maximum value and 0 (yellow) to the minimum value for each variable with a color gradient. Compared to the group with the lowest benefit from retirement (Q1), the group with the highest benefit (Q5) includes more older people (mean age: 65.5 vs. 63.0) and fewer men (% of men: 0.42 vs. 0.57). Asset and income are standardized to z-scores. *P*-values of F-statistics are adjusted using the Bonferroni method.

Analysis of sociodemographic characteristics across CLATO quintiles shown in Fig. 1 revealed distinct patterns. Individuals in the highest CLATO group (Q5) were more likely to be older, female, native-born, and have fewer than three children compared to those in the lowest group (Q1). They also demonstrated higher education levels and greater assets and income. Occupationally, clerks, professionals, and part-time workers were more prevalent in the highest group, whereas service workers, sales personnel, manual laborers, those in physically demanding jobs, and the self-employed were more commonly represented in the lowest group.

**Figure 2.**
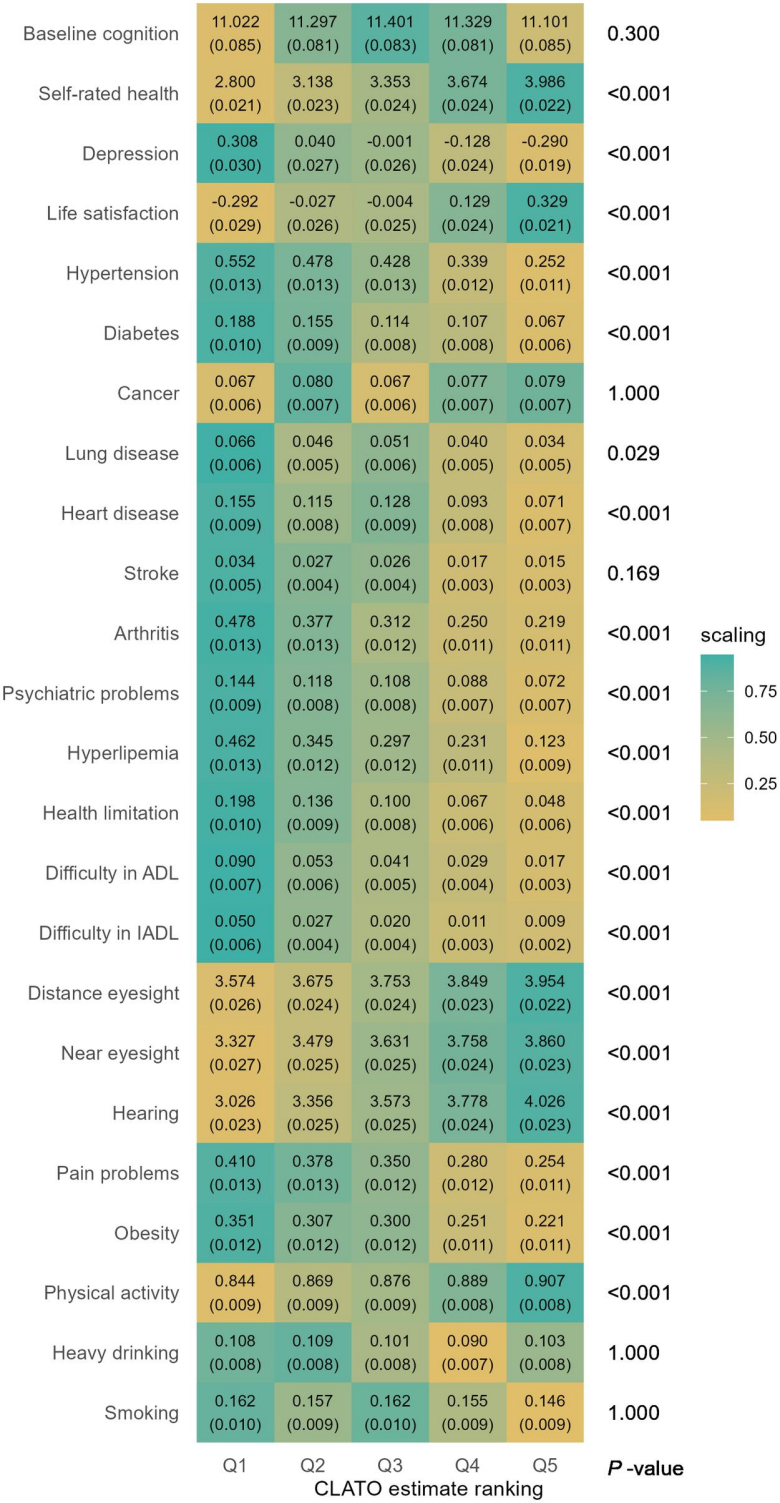
Heterogeneity in health and behaviors. Q1 represents the group with the lowest conditional local average treatment effect on the overlap population (CLATO), whereas Q5 represents the group with the highest CLATO. Each tile indicates the mean value of a covariate within the group, with its standard deviation in parentheses. “Scaling” assigns 1 (green) to the maximum value and 0 (yellow) to the minimum value for each variable with a color gradient. Compared to the group with the lowest benefit from retirement (Q1), the group with the highest benefit (Q5) includes more people with higher self-rated health (mean score: 4.0 vs. 2.8) and fewer people with hypertension (% of having hypertension: 0.25 vs. 0.55). Depression and life satisfaction are standardized to z-scores. *P*-values of F-statistics are adjusted using the Bonferroni method.

Health and well-being metrics also showed variation across CLATO groups (Fig. 2). The highest CLATO group reported better self-rated health, life satisfaction, eyesight, and hearing. Conversely, the lowest CLATO group showed a higher prevalence of various health conditions, including depressive symptoms, hypertension, diabetes, lung and heart diseases, arthritis, psychiatric problems, hyperlipidemia, work limitations, difficulties in activities of daily living (ADL) and instrumental ADL (IADL), and pain-related problems. Obesity was more common in the lowest CLATO group, while regular physical activity was associated with the highest group.

**Figure 3.**
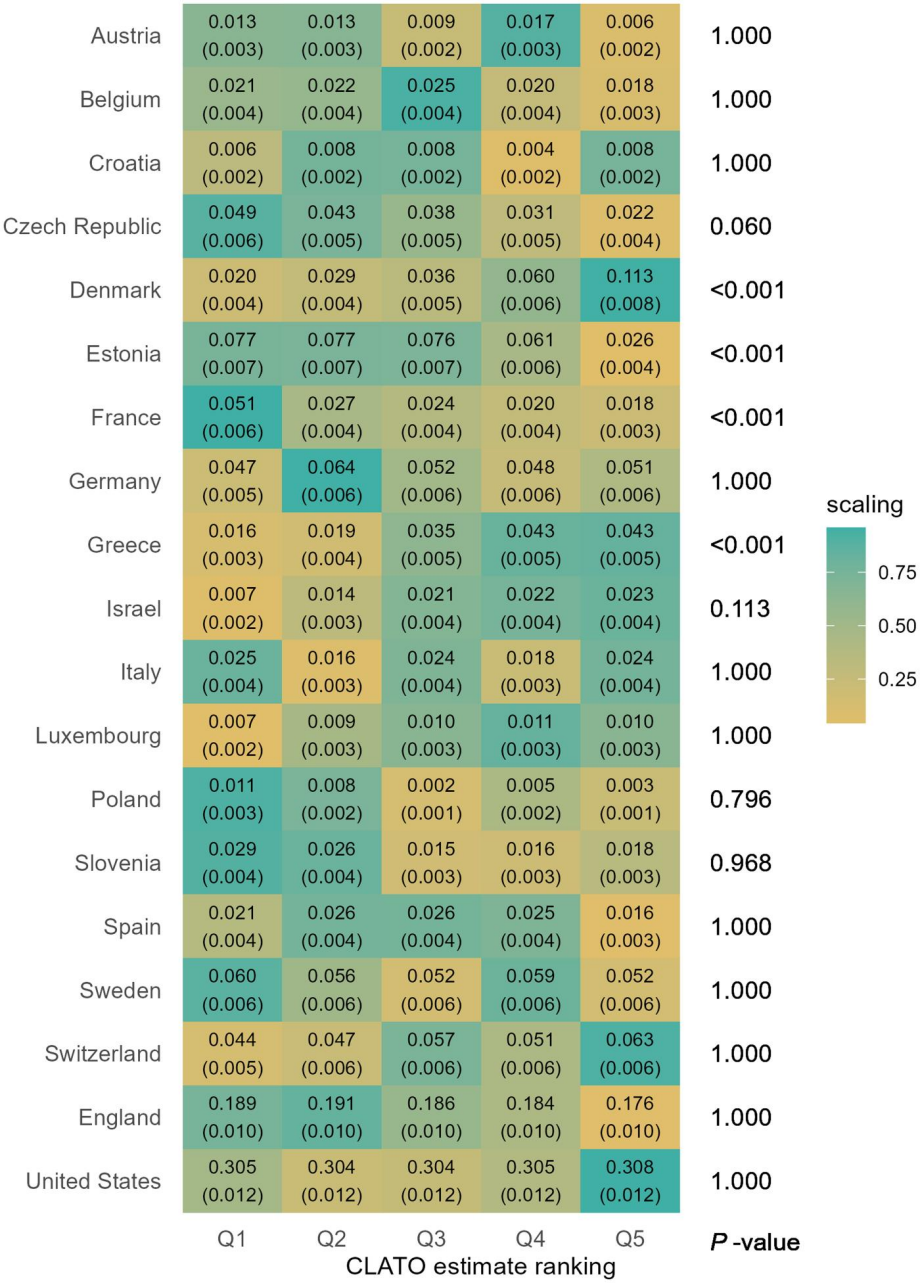
Heterogeneity in countries. Q1 represents the group with the lowest conditional local average treatment effect on the overlap population (CLATO), whereas Q5 represents the group with the highest CLATO. Each tile indicates the mean value of a covariate within the group, with its standard deviation in parentheses. “Scaling” assigns 1 (green) to the maximum value and 0 (yellow) to the minimum value for each variable with a color gradient. Compared to the group with the lowest benefit from retirement (Q1), the group with the highest benefit (Q5) includes more people from Denmark (% of Danish: 0.11 vs. 0.02) and fewer people from France (% of French: 0.02 vs. 0.05). *P*-values of F-statistics are adjusted using the Bonferroni method.

In Fig. 3, geographic analysis revealed that individuals from Denmark and Greece were more likely to fall into higher CLATO groups, whereas those from Estonia and France typically belonged to lower CLATO groups. Age-related analysis showed CLATO estimates increased until age 65 before plateauing. Individuals with average or below-average assets and income showed greater CLATO variations, while those with higher assets and income typically maintained higher CLATO values (Supplementary Fig. S8).

### Sensitivity analyses

Sensitivity analyses maintained the robustness of our findings across various specifications (Supplementary Table S7). These included: restricting the sample to ages 55–75 (Model 1), excluding partially retired individuals (Model 2), analyzing only full-time employees (Model 3), and excluding US data with the largest number of samples (Model 4). ATE estimates remained consistent with our main findings across all specifications.

## Discussion

This study examined the heterogeneous relationship between retirement and cognitive function using harmonized data from 19 countries. Our findings revealed enhanced cognitive function among retirees compared to workers, with conditional ATEs varying according to individual characteristics and countries.

The association observed in our study contrasts with previous research suggesting cognitive decline post-retirement [6, 7, 9, 10], while aligning with studies indicating beneficial associations [15, 16]. The absence of associations in OLS and non-IV forests analyses highlights the importance of methodological choice, suggesting potential negative bias due to health-related selection into retirement. The IV forests method, with its data-driven, nonparametric features, offers greater robustness against model misspecification compared to conventional parametric approaches. The observed improvement of 1.348 words in IV forests represents a substantial effect, corresponding to a 0.42 SD in outcome distribution. According to a systematic review for the effect size of educational interventions on cognition, that of over 0.2 SD is meaningful from a public health perspective [30]. Several mechanisms may explain cognitive enhancement post-retirement. Job strain, a recognized risk factor for cognitive decline [31], is eliminated upon retirement, reducing psychosocial stress. Additionally, retirees can dedicate more time to health-promoting behaviors, such as increased physical activity, improved sleep quality, and smoking cessation [32], which are protective against cognitive decline.

The association between retirement and cognitive function demonstrated notable heterogeneity. Women showed greater cognitive benefits from retirement than men, consistent with evidence indicating women’s higher likelihood of maintaining physical and mental health through post-retirement exercise [17, 20]. Similarly, individuals who exercised before retirement were more likely to experience cognitive benefits, suggesting the importance of pre-existing health behaviors maintained into retirement.

Our findings align with the Grossman model [3], particularly regarding socioeconomic factors. Individuals with higher education, assets, and income demonstrated greater retirement benefits. The model suggests that while retirees have more time but reduced budgets for health investments, those with high socioeconomic status can better afford cognitive health investments. Additionally, better pre-retirement health correlated with enhanced post-retirement cognitive function, consistent with the model’s prediction that healthier individuals have more time for health investments. This relationship between pre-retirement health limitations and post-retirement cognitive function supports previous empirical research [33].

Occupational characteristics influenced retirement outcomes. Professionals showed greater cognitive benefits compared to those retiring from manual labor and physically demanding jobs, supporting previous research linking retirement from complex, mentally demanding occupations to slower cognitive decline [18, 19]. Conversely, retirement from physically demanding jobs showed fewer cognitive benefits, aligning with studies associating such occupations with increased dementia risk [34, 35]. This pattern may reflect the combination of high physical demands with low job control, resulting in reduced cognitive stimulation [14], suggesting workplace cognitive stimulation influences post-retirement cognitive outcomes.

Several limitations warrant consideration. First, our analysis focused on short-term effects, measuring cognitive function two years post-retirement. While previous SHARE research showed early retirement benefits, it also indicated potential negative effects of late [16]. Further research on long-term effects is needed. Second, our heterogeneity analysis was exploratory, with potential confounding between variables, such as education and occupation. Third, despite including 60 candidate variables, unmeasured factors like traumatic brain injury, social isolation, and air pollution might influence retirement’s effect on cognitive function [36]. Fourth, self-reported data may introduce measurement errors though word recall tests have demonstrated predictive validity for dementia onset [37]. Fifth, findings may not generalize beyond Western countries, highlighting the need for comparable studies in rapidly aging Asian nations. Sixth, despite expert harmonization, inter-survey discrepancies may persist. Seventh, our causal inference relies on the validity of the IV. Although the SPA has been widely used as an IV for retirement [4, 5], the assumption of exclusion restriction is not testable. The SPA makes individuals anticipate retirement and may affect their behaviors and mental health, such as starting exercise, undergoing preventive care, or experiencing financial stress. A study using data from the SHARE demonstrated that individuals planning to retire in the next two years had a reduced risk of depression compared with other workers [26]. If such changes in behaviors and mental health before anticipated retirement affect cognitive function, the exclusion restriction assumption can be violated. However, the overidentification test in our 2SLS estimation did not show evidence of its violation.

In conclusion, our findings demonstrate that retirement’s impact on cognitive function varies with individual characteristics and countries. This heterogeneity suggests policymakers should consider incorporating flexible early retirement options into pension systems, allowing individuals to make retirement decisions based on personal circumstances. A machine-learning-based prediction tool could enable individuals to determine optimal retirement timing by evaluating their specific characteristics.

## Ethics approval

Our study used publicly available data that have obtained with informed consent from all participants and ethical approval from relevant local ethics committees. Thus, the Ethics Committee of Kyoto University exempted this study from review.

## Supplementary Material

dyaf201_Supplementary_Data

## Data Availability

The harmonized datasets are available through the Gateway to Global Aging Data website (https://g2aging.org/).

## References

[1] Albert MS , DeKosky ST, Dickson D et al The diagnosis of mild cognitive impairment due to Alzheimer’s disease: recommendations from the National Institute on Aging-Alzheimer’s Association workgroups on diagnostic guidelines for Alzheimer’s disease. Alzheimers Dement 2011;7:270–9. 21514249 10.1016/j.jalz.2011.03.008 PMC3312027

[2] World Health Organization. *Global Action Plan on the Public Health Response to Dementia 2017–2025*. WHO, 2017.

[3] Grossman M. On the concept of health capital and the demand for health. J Polit Econ 1972;80:223–55.

[4] Garrouste C , Perdrix E. Is there a consensus on the health consequences of retirement? A literature review. J Econ Surv 2022;36:841–79.

[5] Nishimura Y , Oikawa M, Motegi H. What explains the difference in the effect of retirement on health? Evidence from global aging data. J Econ Surv 2018;32:792–847.

[6] Bonsang E , Adam S, Perelman S. Does retirement affect cognitive functioning? J Health Econ 2012;31:490–501. 22538324 10.1016/j.jhealeco.2012.03.005

[7] Ebeid M , Oguzoglu U. Short-term effect of retirement on health: evidence from nonparametric fuzzy regression discontinuity design. Health Econ 2023;32:1323–43. 10.1002/hec.4669 36862580

[8] Coe NB , von Gaudecker H-M, Lindeboom M et al The effect of retirement on cognitive functioning. Health Econ 2012;21:913–27. 21818822 10.1002/hec.1771

[9] Behncke S. Does retirement trigger ill health? Health Econ 2012;21:282–300. 21322085 10.1002/hec.1712

[10] Bingley P , Martinello A. Mental retirement and schooling. Eur Econ Rev 2013;63:292–8.

[11] Coe NB , Zamarro G. Retirement effects on health in Europe. J Health Econ 2011;30:77–86. 21183235 10.1016/j.jhealeco.2010.11.002 PMC3972912

[12] Mazzonna F , Peracchi F. Unhealthy retirement? J Human Resources 2017;52:128–51.

[13] Rose L. Retirement and health: evidence from England. J Health Econ 2020;73:102352. 32629223 10.1016/j.jhealeco.2020.102352

[14] Romero Starke K , Seidler A, Hegewald J et al Retirement and decline in episodic memory: analysis from a prospective study of adults in England. Int J Epidemiol 2019;48:1925–36. 31280313 10.1093/ije/dyz135 PMC6929525

[15] Bianchini L , Borella M. Retirement and memory in Europe. Ageing Soc 2016;36:1434–58.

[16] Celidoni M , Dal Bianco C, Weber G. Retirement and cognitive decline. A longitudinal analysis using SHARE data. J Health Econ 2017;56:113–25. 29040897 10.1016/j.jhealeco.2017.09.003

[17] Atalay K , Barrett GF, Staneva A. The effect of retirement on elderly cognitive functioning. J Health Econ 2019;66:37–53. 31108435 10.1016/j.jhealeco.2019.04.006

[18] Carr DC , Willis R, Kail BL et al Alternative retirement paths and cognitive performance: exploring the role of preretirement job complexity. Gerontologist 2020;60:460–71. 31289823 10.1093/geront/gnz079 PMC7117620

[19] Vélez-Coto M , Andel R, Pérez-García M et al Complexity of work with people: associations with cognitive functioning and change after retirement. Psychol Aging 2021;36:143–57. 33764095 10.1037/pag0000584

[20] Sato K , Noguchi H. Heterogeneous associations of retirement with health and behaviors: a longitudinal study in 35 countries. Am J Epidemiol 2025;kwaf126. 10.1093/aje/kwaf126 PMC13017461 40512649

[21] Athey S , Tibshirani J, Wager S. Generalized random forests. Ann Stat 2019;47:1148–78.

[22] Lee J , Phillips D, Wilkens J.; Gateway to Global Aging Data Team. Gateway to Global Aging Data: resources for cross-national comparisons of family, social environment, and healthy aging. J Gerontol B Psychol Sci Soc Sci 2021;76:S5–S16. 33861849 10.1093/geronb/gbab050 PMC8186854

[23] Tulving E. Episodic memory: from mind to brain. Annu Rev Psychol 2002;53:1–25. 11752477 10.1146/annurev.psych.53.100901.135114

[24] Morris JC , Heyman A, Mohs RC et al The Consortium to Establish a Registry for Alzheimer’s Disease (CERAD). Part I. Clinical and neuropsychological assessment of Alzheimer’s disease. Neurology 1989;39:1159–65. 2771064 10.1212/wnl.39.9.1159

[25] Sato K , Noguchi H, Inoue K et al Retirement and cardiovascular disease: a longitudinal study in 35 countries. Int J Epidemiol 2023;52:1047–59. 37155837 10.1093/ije/dyad058 PMC10396426

[26] Vo TT , Phu-Duyen TT. Mental health around retirement: evidence of Ashenfelter’s dip. Glob Health Res Policy 2023;8:35. 37620953 10.1186/s41256-023-00320-3 PMC10464218

[27] Robinson P. Root-N-consistent semiparametric regression. Econometrica 1988;56:931–54.

[28] Mayer M , missRanger: fast imputation of missing values. https://mayer79.github.io/missRanger/2021 (2 January 2023, date last accessed)

[29] Crump RK , Hotz VJ, Imbens GW et al Dealing with limited overlap in estimation of average treatment effects. Biometrika 2009;96:187–99.

[30] Kraft MA. Interpreting effect sizes of education interventions. Educ Res 2020;49:241–53.

[31] Agbenyikey W , Karasek R, Cifuentes M et al Job strain and cognitive decline: a prospective study of the framingham offspring cohort. Int J Occup Environ Med 2015;6:79–94. 25890602 10.15171/ijoem.2015.534 PMC5282587

[32] Xue B , Head J, McMunn A. The impact of retirement on cardiovascular disease and its risk factors: a systematic review of longitudinal studies. The Gerontologist 2020;60:e367–77. 31091304 10.1093/geront/gnz062 PMC7362617

[33] Denier N , Clouston SAP, Richards M et al Retirement and cognition: a life course view. Adv Life Course Res 2017;31:11–21. 28781588 10.1016/j.alcr.2016.10.004 PMC5539912

[34] Nabe-Nielsen K , Holtermann A, Gyntelberg F et al The effect of occupational physical activity on dementia: results from the Copenhagen Male Study. Scand J Med Sci Sports 2021;31:446–55. 33038033 10.1111/sms.13846

[35] Zotcheva E , Bratsberg B, Strand BH et al Trajectories of occupational physical activity and risk of later-life mild cognitive impairment and dementia: the HUNT4 70+ study. Lancet Reg Health Eur 2023;34:100721. 37927437 10.1016/j.lanepe.2023.100721 PMC10625024

[36] Livingston G , Huntley J, Liu KY et al Dementia prevention, intervention, and care: 2024 report of the Lancet standing Commission. Lancet Lond Engl 2024;404:572–628. 10.1016/S0140-6736(24)01296-0 39096926

[37] Tierney MC , Moineddin R, McDowell I. Prediction of all-cause dementia using neuropsychological tests within 10 and 5 years of diagnosis in a community-based sample. J Alzheimers Dis 2010;22:1231–40. 20930315 10.3233/JAD-2010-100516

