# Supplementary material for: Heterogeneity in the association between retirement and cognitive function: a machine learning analysis across 19 countries
Source: Int J Epidemiol. 2025 Nov 24;54(6):dyaf201. doi: 10.1093/ije/dyaf201 (PMC12641609; doi:10.1093/ije/dyaf201)
Supplement: dyaf201_Supplementary_Data [file dyaf201_supplementary_data.pdf]

Supplementary Materials

Heterogeneity in the Association Between Retirement and Cognitive Function: A Machine Learning Analysis Across 19 Countries

Contents

Figure S1. Sample Flowchart 2
Table S1. Comparison of Characteristics Between Included and Excluded Individuals 3
Method S1. Measurement of Labor Force Status 5
Table S2. Categories of Labor Force Status 6
Table S3. Early and Official Retirement Age 6
Figure S2. Retirement Rate of Men 7
Figure S3. Retirement Rate of Women 8
Method S2. IV Forests Algorithm 9
Table S4. Definition of Covariates 11
Table S5. Occupational Codes 13
Table S6. Number of Imputed Values 15
Figure S4. Distribution of Cognitive Function 16
Figure S5. Variable Importance 16
Figure S6. Distribution of Conditional Average Treatment Effects 17
Figure S7. Calibration Plot for CLATO 18
Figure S8. Partial Dependence Plot for Continuous Variables 19
Table S7. Sensitivity Analyses 20
Supplementary References 20

Figure S1. Sample Flowchart

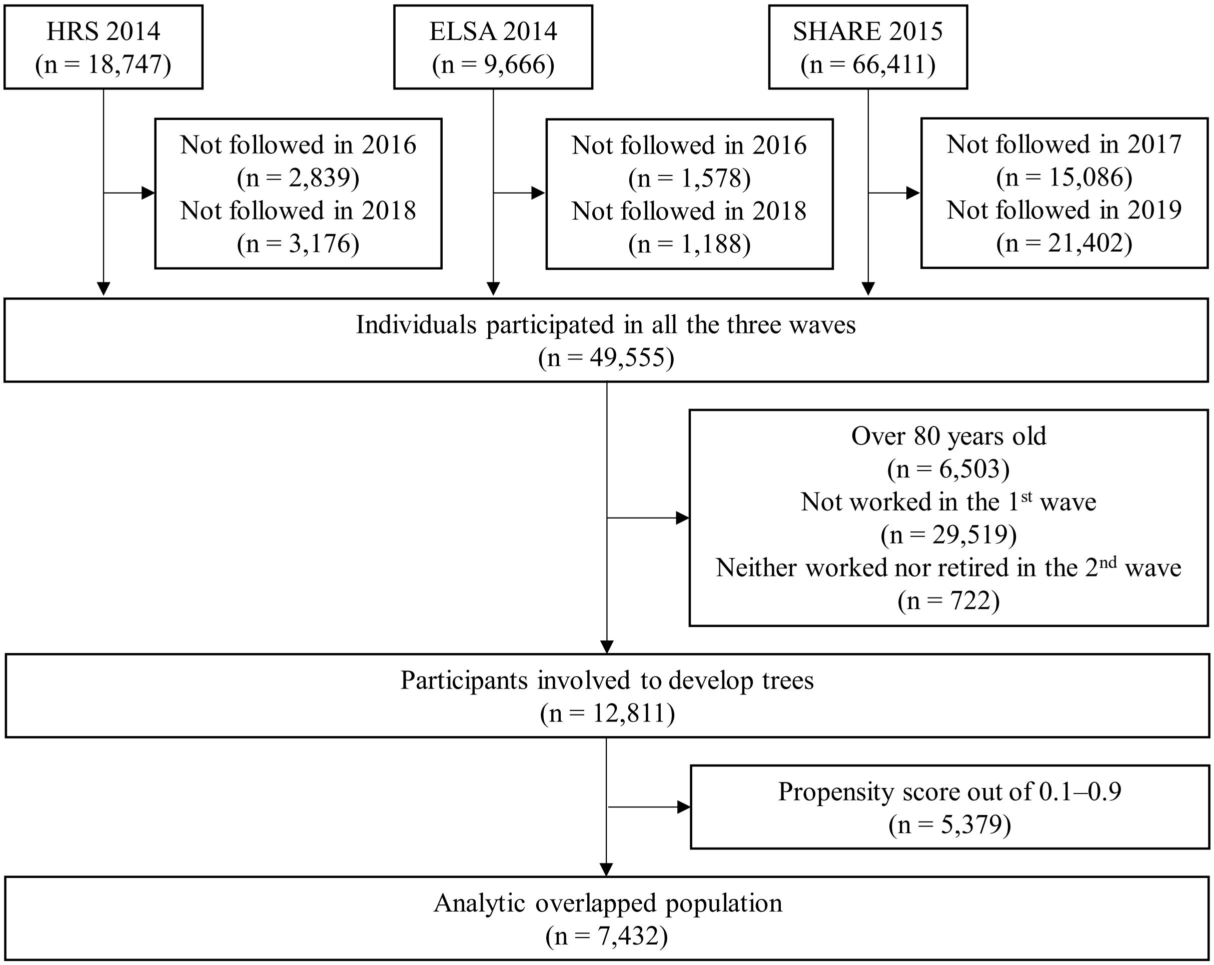

HRS stands for the Health and Retirement Study, ELSA stands for the English Longitudinal Study on Ageing, and SHARE stands for the Survey of Health, Ageing and Retirement in Europe.

Table S1. Comparison of Characteristics Between Included and Excluded Individuals

Variables, n (%)	Included Worker<br>n = 10 438	Included Retiree<br>n = 2373	Excluded<br>n = 12 033
Sociodemographic Characteristics			
Age, year, mean (SD)	57.7 (4.91)	62.6 (4.74)	56.6 (5.89)
Men	4873 (46.7)	1157 (48.8)	5558 (46.2)
Foreign-born	1362 (13.1)	248 (10.5)	1741 (14.5)
Education, mean (SD)	2.2 (0.67)	2.1 (0.69)	2.1 (0.72)
Married	8339 (79.9)	1826 (77.0)	9876 (82.1)
Living alone	1536 (14.7)	437 (18.4)	1566 (13.0)
No children	1071 (10.3)	230 (9.7)	1237 (10.3)
≥3 children	3651 (35.0)	819 (34.6)	3744 (31.2)
Asset, z-score, mean (SD)	0.0 (1.02)	0.1 (0.81)	0.0 (1.01)
Income, z-score, mean (SD)	0.1 (0.96)	0.0 (0.90)	-0.1 (1.05)
Professional	3823 (41.4)	853 (40.8)	3852 (37.6)
Clerk	1427 (15.5)	321 (15.3)	1524 (14.9)
Service & sales	1894 (20.5)	425 (20.3)	2026 (19.8)
Manual labor	2084 (22.6)	494 (23.6)	2842 (27.7)
Physical demand, mean (SD)	2.3 (1.08)	2.3 (1.05)	2.4 (1.06)
Part-time job	2428 (23.7)	806 (34.7)	2807 (23.8)
Self-employed	1802 (17.3)	389 (16.4)	2108 (17.6)
Health & Behaviors			
Baseline cognition, mean (SD)	11.5 (3.14)	11.0 (3.28)	11.0 (3.27)
Self-rated health, mean (SD)	3.4 (0.97)	3.3 (0.96)	3.3 (0.99)
Depression, z-score, mean (SD)	0.0 (0.97)	0.0 (0.95)	0.0 (1.03)
Life satisfaction, z-score, mean (SD)	0.0 (0.97)	0.1 (0.96)	0.0 (1.03)
Hypertension	3486 (33.4)	1055 (44.5)	3862 (32.1)
Diabetes	1008 (9.7)	331 (13.9)	1054 (8.8)
Cancer	619 (5.9)	193 (8.1)	622 (5.2)
Lung disease	419 (4.0)	127 (5.4)	538 (4.5)
Heart disease	801 (7.7)	289 (12.2)	881 (7.3)
Stroke	171 (1.6)	74 (3.1)	238 (2.0)
Arthritis	2665 (25.5)	856 (36.1)	2638 (21.9)
Psychiatric problems	1012 (9.7)	272 (11.5)	1070 (8.9)
Hyperlipemia	2575 (24.7)	683 (28.8)	2311 (19.2)
Health limitation in working	1031 (10.0)	316 (13.5)	1374 (11.6)
Difficulty in ADL	363 (3.5)	145 (6.1)	402 (3.3)
Difficulty in IADL	209 (2.0)	51 (2.1)	205 (1.7)
Distance eyesight, mean (SD)	3.8 (0.94)	3.8 (0.92)	3.8 (0.94)
Near eyesight, mean (SD)	3.6 (1.00)	3.6 (0.98)	3.6 (1.00)
Hearing, mean (SD)	3.7 (0.99)	3.5 (1.00)	3.7 (0.98)
Pain problems	3282 (31.7)	857 (36.4)	3994 (33.6)
Obesity	2632 (27.1)	656 (29.2)	2487 (21.9)
Physical activity	9343 (89.6)	2060 (86.9)	10792 (89.8)
Heavy drinking	1001 (9.8)	253 (11.0)	1204 (10.2)
Smoking	1770 (17.6)	392 (17.2)	2644 (23.3)
Countries			
Austria	126 (1.2)	41 (1.7)	334 (2.8)
Belgium	364 (3.5)	62 (2.6)	1129 (9.4)
Croatia	141 (1.4)	18 (0.8)	334 (2.8)

Czech Republic	270 (2.6)	131 (5.5)	477 (4.0)
Denmark	688 (6.6)	94 (4.0)	780 (6.5)
Estonia	740 (7.1)	97 (4.1)	999 (8.3)
France	354 (3.4)	97 (4.1)	476 (4.0)
Germany	704 (6.7)	131 (5.5)	604 (5.0)
Greece	320 (3.1)	59 (2.5)	684 (5.7)
Israel	136 (1.3)	31 (1.3)	362 (3.0)
Italy	360 (3.4)	46 (1.9)	790 (6.6)
Luxembourg	134 (1.3)	38 (1.6)	194 (1.6)
Poland	96 (0.9)	14 (0.6)	266 (2.2)
Slovenia	232 (2.2)	67 (2.8)	355 (3.0)
Spain	215 (2.1)	62 (2.6)	753 (6.3)
Sweden	392 (3.8)	152 (6.4)	487 (4.0)
Switzerland	470 (4.5)	117 (4.9)	353 (2.9)
England	1679 (16.1)	436 (18.4)	900 (7.5)
United States	3017 (28.9)	680 (28.7)	1756 (14.6)

Excluded individuals were aged under 78 years and worked in the first wave.

Method S1. Measurement of Labor Force Status

The Health and Retirement Study (HRS)—Participants in HRS provided information on their labor force status at several time points in an interview. First, HRS asks the participants to select all applicable options from a list that includes 1) working now, 2) unemployed and looking for work, 3) temporarily laid off, on sick or other leave, 4) disabled, 5) retired, 6) homemaker, or 7) other (specify). It also asks them whether they are currently working for payment, the usual number of hours per week if applicable, and whether they consider themselves partly retired, completely retired, or not retired.

If the participant reports working full-time (i.e. working 35+ hours per week or 36+ weeks per year), the harmonized variable is set to "working full-time". If the participant is working part-time and does not mention retirement, it is set to "working part-time". If the participant is working part-time and mentions retirement, it is set to "partly retired". If the participant is not working but is looking for a job, it is set to "unemployed". If the participant is not looking for a job and there is any mention of retirement, it is set to "retired". If retirement is not mentioned and disabled employment status is given, it is set to "disabled". Otherwise, the variable is set to "not in the labor force".

The English Longitudinal Study on Ageing (ELSA)—ELSA asks participants, "Which of these, would you say, best describes your situation?" They then choose the best description of their current labor force status from a list of options: 1) employed, 2) self-employed, 3) unemployed, 4) partly retired, 5) retired, 6) permanently sick or disabled, or 7) looking after home or family. The harmonized variable was constructed based on responses to this direct question.

The Survey of Health, Ageing and Retirement in Europe (SHARE)—SHARE asks participants, "In general, how would you describe your current situation?" They then choose the best description of their current labor force status from a list of options: 1) retired, 2) employed or self-employed (including working for a family business), 3) unemployed and looking for work, 4) permanently sick or disabled, 5) homemaker, or 6) other (renter, living off own property, student, or doing voluntary work). The harmonized variable was constructed based on responses to this direct question.

Table S2. Categories of Labor Force Status

This study	HRS	ELSA	SHARE
Included as workers	1. working full-time	1. employed	1. employed or self-employed
	2. working part-time	2. self-employed	
Included as retirees	4. partly retired	4. partly retired	5. retired
	5. retired	5. retired	
Excluded from analyses	3. unemployed	3. unemployed	3. unemployed
	6. disabled	6. disabled	6. permanently sick or disabled
	7. not in the labor force	7. looking after home or family	8. homemaker

HRS stands for the Health and Retirement Study, ELSA stands for the English Longitudinal Study on Ageing, and SHARE stands for the Survey of Health, Ageing and Retirement in Europe. Retirement status was determined based on the variable of labor force status (RwLBRF) in the harmonized datasets. "Partly retired" includes those who self-identified as a retiree but engaged in a part-time job (see Supplementary Method S1 for details).

Table S3. Early and Official Retirement Age

Country	Year	Men		Women	
		ERA	ORA	ERA	ORA
Austria	2017	NA	65	NA	60
Belgium	2017	62.5	65	62.5	65
Croatia	2017	60	65	56.75	61.75
Czech Republic[a]	2017	60	63.17	59.33	62.33
Denmark	2017	NA	65	NA	65
England	2016	NA	65	NA	63
Estonia	2017	60	63	60	63
France	2017	62	67	62	67
Germany	2017	63	65.5	63	65.5
Greece	2017	62	67	62	67
Israel	2017	NA	67	NA	62
Italy	2017	62	66.58	62	66.58
Luxembourg	2017	57	65	57	65
Poland	2017	NA	65	NA	60
Slovenia	2017	59.67	65	59.33	63.5
Spain	2017	61.42	65.42	61.42	65.42
Sweden	2017	61	65	61	65
Switzerland	2017	63	65	62	64
United States	2016	62	66	62	66

ERA and ORA stand for early and official retirement age, respectively. NA denotes not applicable.
Source: The United States Social Security Administration "Social Security Programs Throughout the World"; OECD "Pensions at a Glance"; websites of the authorities of each country.
[a] ORA for women is determined according to the number of raised children. ORA of 62 years and 4 months is for women without children in 2017.

Figure S2. Retirement Rate of Men

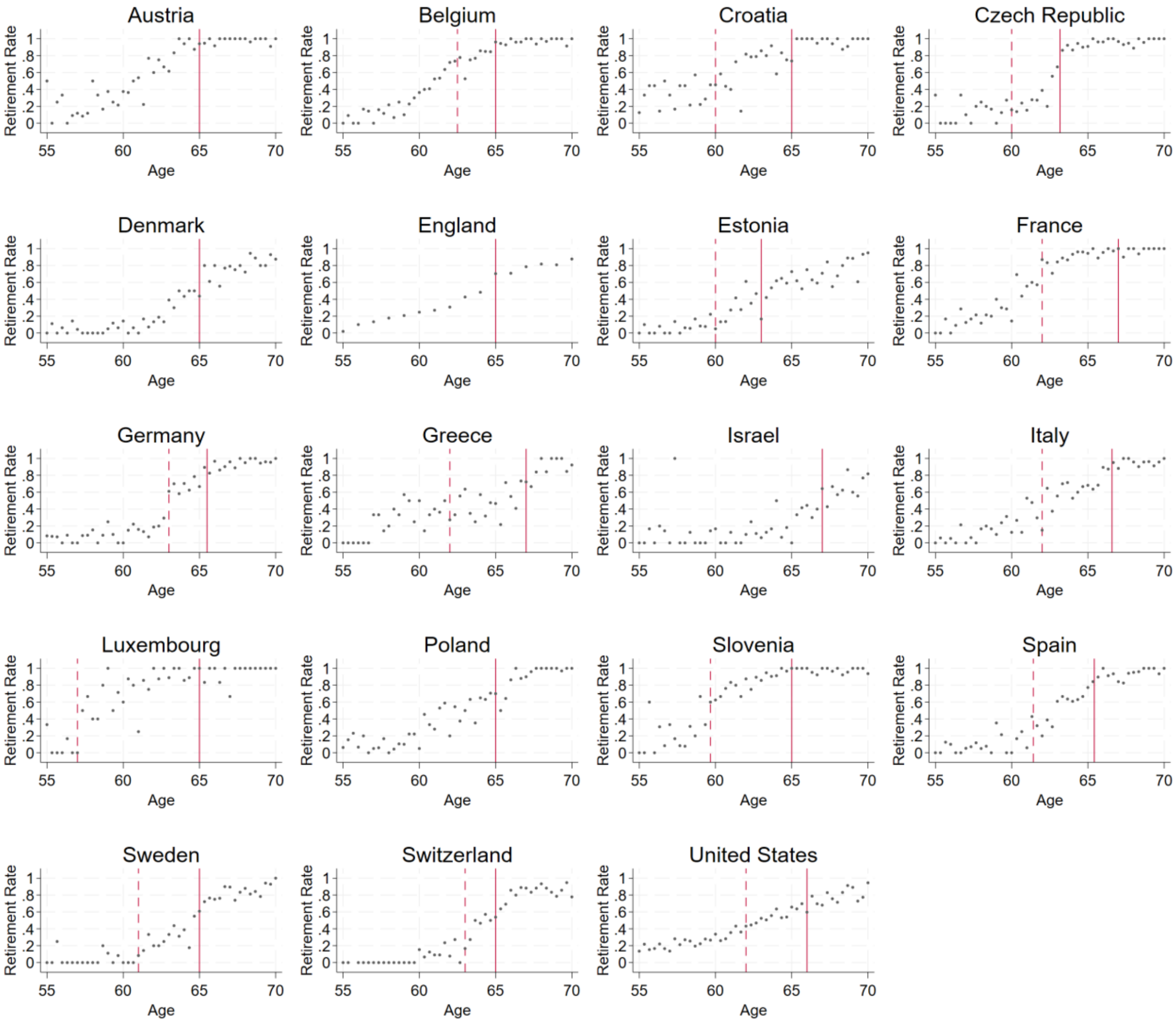

Each dot in the figure represents the average retirement rate for each 4-month interval (due to unavailable monthly age data in England). The retirement rate is calculated by dividing the number of retirees by the sum of retirees and workers within each interval. The dashed red line denotes the early retirement age, while the solid red line denotes the official retirement age in the year of the second wave survey.

Figure S3. Retirement Rate of Women

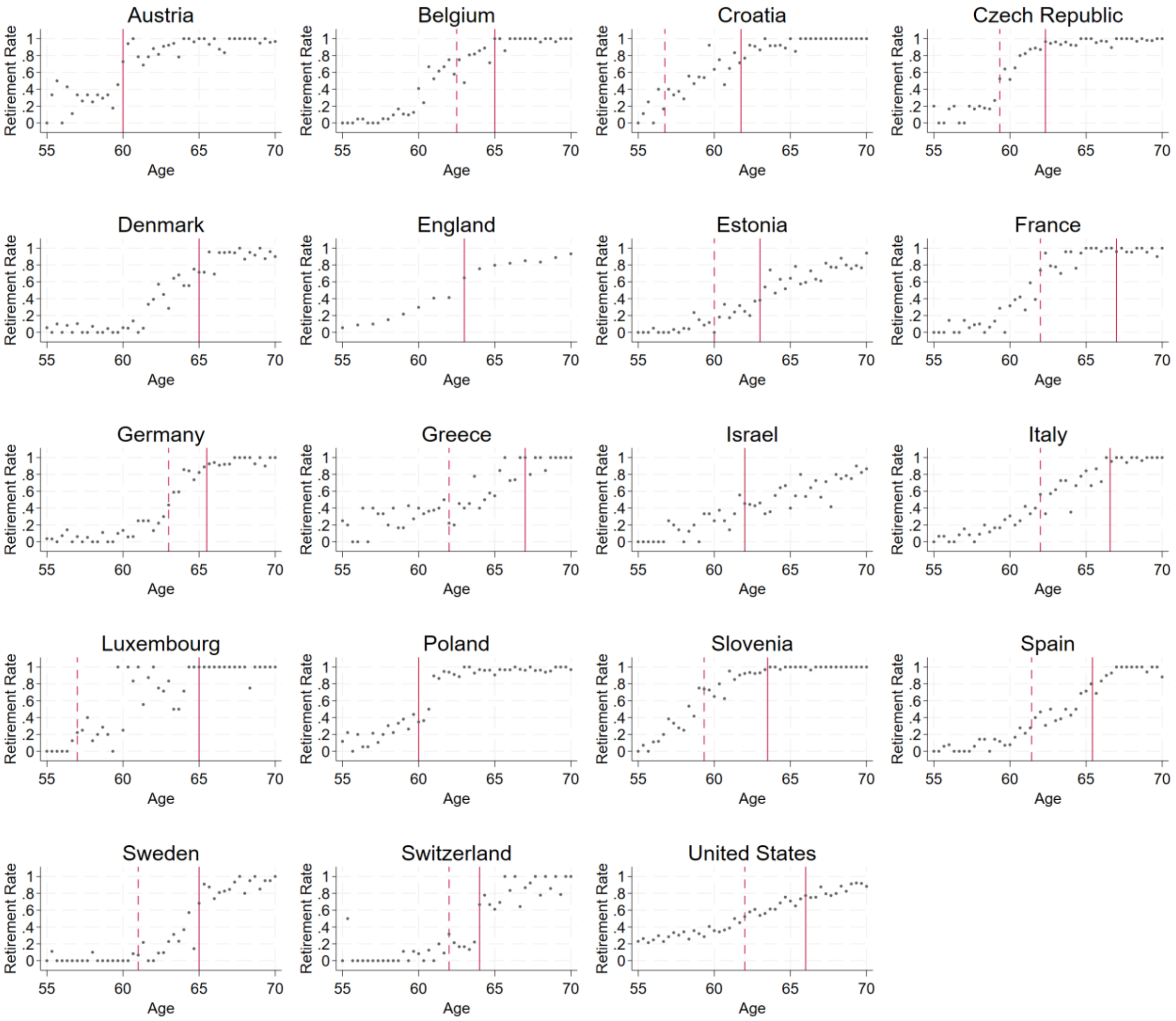

Each dot in the figure represents the average retirement rate for each 4-month interval (due to unavailable monthly age data in England). The retirement rate is calculated by dividing the number of retirees by the sum of retirees and workers within each interval. The dashed red line denotes the early retirement age, while the solid red line denotes the official retirement age in the year of the second wave survey.

Method S2. IV Forests Algorithm

To assess the heterogeneous treatment effect of retirement, we used an IV forests algorithm developed by Athey and colleagues [1]. Let n samples indexed by $i = 1, \dots, n$ assumed to be independent and identically distributed. Each observations $O_i = \{Y_i, W_i, Z_i\}$ consists of an outcome $Y_i \in \mathbb{R}$ (cognitive function), a treatment assignment $W_i \in \{0,1\}$ (retirement), and an IV $Z_i \in \{0,1\}$ (SPA), alongside a set of auxiliary covariates $X_i \in \mathcal{X}$. The conditional effects of interest $\theta(x)$ are solutions to the local moment conditions:

$$\mathbb{E}\left[\psi_{\theta(x),\nu(x)}(O_i) \mid X_i = x\right] = 0 \quad \forall\, x \in \mathcal{X},$$

where $\psi(\cdot)$ is a scoring function and $\nu(x)$ is an optional nuisance parameter. The IV forests estimate $\left(\hat{\theta}(x), \hat{\nu}(x)\right)$ are obtained by solving:

$$\left(\hat{\theta}(x), \hat{\nu}(x)\right) \in \arg\min_{\theta,\nu} \left\{ \left\| \sum_{i=1}^{n} \alpha_i(x) \psi_{\theta,\nu}(O_i) \right\|_2 \right\},$$

assuming a unique root exists, where $\sum_{i=1}^{n} \alpha_i(x) \psi_{\hat{\theta}(x),\hat{\nu}(x)}(O_i) = 0$. IV forests incorporate similarity weights $\alpha_i(x)$ derived from random forests with B trees, indexed by $b = 1, \dots, B$

$$\alpha_{bi}(x) = \frac{1(\{X_i \in L_b(x)\})}{|L_b(x)|}, \qquad \alpha_i(x) = \frac{1}{B} \sum_{b=1}^{B} \alpha_{bi}(x).$$

Here, $L_b(x)$ denotes the set of training samples falling in the same leaf as target sample x in tree b, and $\alpha_i(x)$ represents the frequency of the ith training sample falling into the same leaf as x. The forests-based algorithm partitions training samples to maximize the squared difference in treatment effect estimates across leaves (i.e., heterogeneity), ensuring $\alpha_i(x)$ fits $\theta(x)$ well. Estimates $\hat{\theta}_x$ exhibit asymptotic normality using an "honesty" subsampling technique [1–3]. The "honesty" approach involves dividing the sample into three subsets; the "splitting" subset for partitioning samples and tree development, the "estimation" subset for leaf-specific treatment effect estimation, and the "test" subset for validation.

To apply the forests-based algorithm to an IV regression, Athey and colleagues assume a structural model:

$$Y_i = \mu(X_i) + \tau(X_i) W_i + \varepsilon_i$$

where $\mu(X_i)$ denotes a nuisance intercept parameter; $\tau(X_i)$ is interpreted as the causal effect of W_i on Y_i, and ε_i is an error term potentially correlated with W_i. To ensure consistency of $\tau(X_i)$ despite W_i and ε_i correlation, an IV Z_i is used. If Z_i is independent of ε_i conditional on X_i, and the covariance of Z_i and W_i conditional on X_i is nonzero, $\tau(X_i)$ is identified as:

$$\tau(X_i) = \frac{\mathrm{Cov}[Y_i, Z_i \mid X_i = x]}{\mathrm{Cov}[W_i, Z_i \mid X_i = x]}.$$

In this setting, the scoring function $\psi(\cdot)$ is defined as:

$$\psi_{\tau(x),\mu(x)}(Y_i, W_i, Z_i) = \begin{bmatrix} Z_i(Y_i - W_i \tau(x) - \mu(x)) \\ Y_i - W_i \tau(x) - \mu(x) \end{bmatrix}.$$

Then, $\tau(x)$ is estimated via moment functions $\mathbb{E}\left[Z_i\left(Y_i - W_i\tau(x) - \mu(x)\right)\middle|X_i = x\right] = 0$ and $\mathbb{E}[Y_i - W_i\tau(x) - \mu(x)|X_i = x] = 0$. Biewen and Kugler [4] extend IV forests to a multiple IVs setting (where Z_i is a $M \times 1$ vector) by defining $\psi(\cdot)$ as:

$$\psi_{\tau(x),\mu(x),\gamma_1(x),\gamma_0(x)}(Y_i, W_i, Z_i) = \begin{bmatrix} \dot{W}_i(Y_i - \dot{W}_i\tau(x) - \mu(x)) \\ Y_i - \dot{W}_i\tau(x) - \mu(x) \\ Z_i(W_i - Z_i'\gamma_1(x) - \gamma_0(x)) \\ W_i - Z_i'\gamma_1(x) - \gamma_0(x) \end{bmatrix},$$

where $\dot{W}_i = \gamma_0(x) + Z_i'\gamma_1(x)$. Estimates of a conditional local average treatment effect $\hat{\tau}(x)$ are derived from solving $M + 3$ moment conditions, complemented by weights $\alpha_i(x)$.

We employed the IV forests method with 2000 trees, optimizing their parameters through cross-validation. To assess the effect of retirement on cognitive function using IV forests, we estimated the local average treatment effect on the overlap population (LATO). We define the conditional mean of the outcome as $y(x) = \mathbb{E}[Y_i|X_i = x]$, the propensity score of the treatment as $w(x) = \mathbb{E}[W_i|X_i = x]$, and the propensity score of the instrument as $z(x) = \mathbb{E}[Z_i|X_i = x]$. IV forests yield out-of-bag estimates (i.e., the prediction of the ith observation is obtained via trees fitted without using the ith observation) of these marginal expectations, denoted as $\hat{y}^{(-i)}, \hat{w}^{(-i)}, \hat{z}^{(-i)}$, which achieve $\sqrt{n}$ consistency using a machine-learning approach [5]. Subsequently, we restricted samples to the overlap population $\mathcal{P}$ by truncating the estimated treatment propensity score $\hat{w}^{(-i)}(x)$ to value between 0.1 and 0.9 [6]. To obtain the LATO through non-parametric estimation, we computed conditionally centered outcomes $\tilde{Y}_i = Y_i - \hat{y}^{(-i)}(X_i)$, $\widetilde{W}_i = W_i - \hat{w}^{(-i)}(X_i)$, and $\tilde{Z}_i = Z_i - \hat{z}^{(-i)}(X_i)$, followed by a residual-on-residual two-stage least squares (2SLS) regression using these centered outcomes $\{\tilde{Y}_i, \widetilde{W}_i, \tilde{Z}_i\}_{i=1}^{n} \in \mathcal{P}$ [7].

To assess the heterogeneity captured by IV forests, we constructed calibration plots based on the ranking of estimated conditional local average treatment effect on the overlap population (CLATO) $\hat{\tau}(x)$. To ensure valid inference of $\hat{\tau}(X_i)$, using the “honesty” property, we employed a ten-fold cross-validation approach: fitting IV forests on nine folds and predicting $\hat{\tau}(x)$ on the remaining fold. Observations were categorized into quintile (Q1 [the lowest CLATO; the subgroup of individuals who received the least benefits from retirement] to Q5 [the highest CLATO; the subgroup of individuals who received the most benefits from retirement]) based on the ranking of $\hat{\tau}(x)$ within each fold. This procedure was repeated across iterations to estimate LATO for each quintile subgroup. Furthermore, to assess covariates heterogeneity, we compared the mean values of covariates across these groups and visualized partial dependence plots with the continuous variables on the x-axis and the out-of-bag predictions of CLATO $\hat{\tau}^{(-i)}(X_i)$ on the y-axis.

Table S4. Definition of Covariates

Covariate	Type	Definition
Age	Continuous	This variable is the participant's age in months at the time of the second wave interview.
Men	Binary	This variable is coded as 1 for men and 0 for women.
Foreign-born	Binary	This variable is coded as 1 if the interview did not take place in the country of birth and 0 otherwise.
Education	Ordered	This variable is coded as 1 for less than upper secondary education, 2 for upper secondary and vocational training, and 3 for tertiary education according to the 1997 International Standard Classification of Education.
Married	Binary	This variable is coded as 1 for married or partnered and 0 otherwise.
Living alone	Binary	This variable is coded as 1 for those whose household size is 1 and 0 otherwise.
Number of children	Binary	Based on a variable indicating the participant's number of living children (including natural, foster, adopted, or stepchildren), we created two binary variables; "no children" indicates 1 if the number of children is zero and 0 otherwise; "≥3 children" indicates 1 if the number of children is three or more and 0 otherwise.
Asset	Continuous	This variable is the net value of assets at the couple-level unit calculated as the value of all wealth components (including housing, financial, and non-financial assets) minus that of all debts. To make the variables in different surveys comparable, we standardized them to z-scores for each survey. See Angrisani & Lee [8] for details about the harmonization of wealth measures.
Income	Continuous	This variable is the total income at the couple level including earnings, capital income, pensions, and public transfers. To make the variables in different surveys comparable, we standardized them to z-scores for each survey. See Angrisani & Lee [9] for details about the harmonization of income measures.
Occupation	Binary	We created five binary variables indicating the participant's occupation: professional, clerk, service and sales, and manual labor. We categorized occupations based on the 2010 Census occupations in the HRS, the Standard Occupational Classification (2000) in the ELSA, and the 1988 International Standard Classification of Occupations in the SHARE. See Supplementary Table S5 for details about the occupational codes.
Physical demand	Ordered	This variable is a 4-point Likert scale indicating the degree to which the participant agrees that their job is physically demanding: 1 = strongly disagree; 2 = disagree; 3 = agree; 4 = strongly agree.
Part-time job	Binary	This variable is coded as 1 if the participant works less than 35 hours per week and 0 otherwise.
Self-employed	Binary	This variable is coded as 1 if the participant reports to be self-employed and 0 otherwise.
Baseline cognition	Continuous	This variable indicates baseline cognitive function measured in the same way as the outcome.
Self-rated health	Ordered	This variable is a 5-point Likert scale indicating self-rated health: 1 = poor; 2 = fair; 3 = good; 4 = very good; 5 = excellent.
Depression	Continuous	Higher scores of this variable indicate more severe depression. The HRS and the ELSA use a short version of the Center for Epidemiologic Studies Depression (CES-D) to measure depression, whereas the SHARE uses the EURO-D scale. To make the variables using different measures comparable, we standardized them to z-scores for each survey.

Life satisfaction	Continuous	Higher scores of this variable indicate higher levels of the participant's life satisfaction. The HRS uses a 5-point Likert scale; the ELSA uses a 7-point Likert scale, and the SHARE uses a 10-point Likert scale to measure life satisfaction. The harmonized datasets provide a variable standardized to z-scores for each survey to render them comparable.
Diagnosed diseases	Binary	We have nine variables of chronic medical conditions: hypertension, diabetes, cancer, lung disease, heart disease, stroke, arthritis, psychiatric problems, and hyperlipemia. These variables indicate 1 if a doctor has ever told the participant that they have the conditions and 0 otherwise. See Hu & Lee [10] for details about the harmonization of chronic medical conditions.
Health limitation	Binary	This variable indicates 1 if the participant reports that an impairment or health problem limits the kind or amount of paid work and 0 otherwise.
Difficulty in ADL	Binary	This variable indicates 1 if the participant has difficulties with any of the five ADLs including bathing or showering, dressing, eating, getting in and out of bed, and walking across a room, and 0 otherwise.
Difficulty in IADL	Binary	This variable indicates 1 if the participant has difficulties with any of the five IADLs including using the telephone, managing money, taking medications, shopping for groceries, and preparing a hot meal, and 0 otherwise.
Eyesight and hearing	Ordered	We have three 5-point Likert scales for self-reported distance eyesight, near eyesight, and hearing: 1 = poor; 2 = fair; 3 = good; 4 = very good; 5 = excellent.
Pain problems	Binary	This variable indicates 1 if the participant reports to be troubled with pain and 0 otherwise.
Obesity	Binary	This variable indicates 1 if the participant's body mass index is 30 kg/m^2 or higher and 0 otherwise [11].
Physical activity	Binary	This variable indicates 1 if the participant engages in vigorous or moderate physical activity once or more per week and 0 otherwise.
Heavy drinking	Binary	This variable indicates 1 if the participant reports having 15 or more drinks per week for men and 8 or more drinks for women, and 0 otherwise [12].
Smoking	Binary	This variable indicates 1 if the participant reports smoking now and 0 otherwise.
Countries	Binary	We have 19 binary variables indicating the place of the interview: Austria, Belgium, Croatia, Czech Republic, Denmark, England, Estonia, France, Germany, Greece, Israel, Italy, Luxembourg, Poland, Slovenia, Spain, Sweden, Switzerland, and the United States.

Table S5. Occupational Codes

This study	HRS	ELSA	SHARE
	The 2010 Census	Standard Occupational Classification (2000)	1988 International Standard Classification of Occupations
Professional	1. Management occupations<br>2. Business and financial specialists<br>3. Computer and mathematical occupations<br>4. Architecture and engineering occupations<br>5. Life, physical, and social science occupations<br>6. Community and social services occupations<br>7. Legal occupations<br>8. Education, training, and library occupations<br>9. Arts, design, entertainment, sports, and media occupations<br>10. Healthcare practitioners and technical occupations<br>11. Healthcare support occupations<br>23. Military-specific occupations	1. Managers and senior officials<br>2. Managers and proprietors in agriculture and services<br>3. Science and technology professionals<br>4. Health professionals<br>5. Teaching and research professionals<br>6. Business and public service professionals<br>7. Science and technology associate professionals<br>8. Health and social welfare associate professionals<br>10. Culture, media, and sports occupations<br>11. Business and public service associate professionals	0. Armed forces<br>1. Legislator, senior official, or manager<br>2. Professional<br>3. Technician or associate professional
Clerk	17. Office and administrative support occupations	12. Administrative occupations<br>13. Secretarial and related occupations	4. Clerk
Service & sales	12. Protective service occupations<br>13. Food preparation and serving occupations<br>14. Building and grounds cleaning and maintenance occupations<br>15. Personal care and service occupations<br>16. Sales occupations	9. Protective service occupations<br>18. Caring personal service occupations<br>19. Leisure and other personal service occupations<br>20. Sales occupations<br>21. Customer service occupations<br>25. Elementary administration and service occupations	5. Service worker and shop and market sales worker
Manual labor	18. Farming, fishing, and forestry occupations<br>19. Construction and extraction occupations<br>20. Installation, maintenance, and repair workers<br>21. Production occupations	14. Skilled agricultural trades<br>15. Skilled metal and electrical trades<br>16. Skilled construction and building trades<br>17. Textiles, printing, and other skilled trades	6. Skilled agricultural or fishery worker<br>7. Craft and related trades worker<br>8. Plant and machine operator or assembler<br>9. Elementary occupation

22. Transportation and material moving occupations	22. Process, plant, and machine operatives<br>23. Transport and mobile machine drivers and operatives<br>24. Elementary trades, plant, and storage-related occupations	

HRS stands for the Health and Retirement Study, ELSA stands for the English Longitudinal Study on Ageing, and SHARE stands for the Survey of Health, Ageing and Retirement in Europe.

Table S6. Number of Imputed Values

Variable	Imputed values
Cognitive function	793
Retirement	0
Age	0
Men	0
Foreign-born	8
Education	210
Married	5
Living alone	0
No children	24
≥3 children	24
Asset	66
Income	955
Professional	1490
Clerk	1490
Service & sales	1490
Manual labor	1490
Physical demand	1715
Part-time job	223
Self-employed	13
Baseline cognition	268
Self-rated health	93
Depression	170
Life satisfaction	470
Hypertension	2
Diabetes	2
Cancer	2
Lung disease	2
Heart disease	2
Stroke	2
Arthritis	2
Psychiatric problems	2
Hyperlipemia	8
Health limitations in working	136
Difficulty in ADL	2
Difficulty in IADL	2
Distance eyesight	95
Near eyesight	99
Hearing	8
Pain problems	102
Obesity	844
Physical activity	14
Heavy drinking	331
Smoking	470

ADL and IADL stand for activities of daily living and instrumental activities of daily living, respectively.

Figure S4. Distribution of Cognitive Function

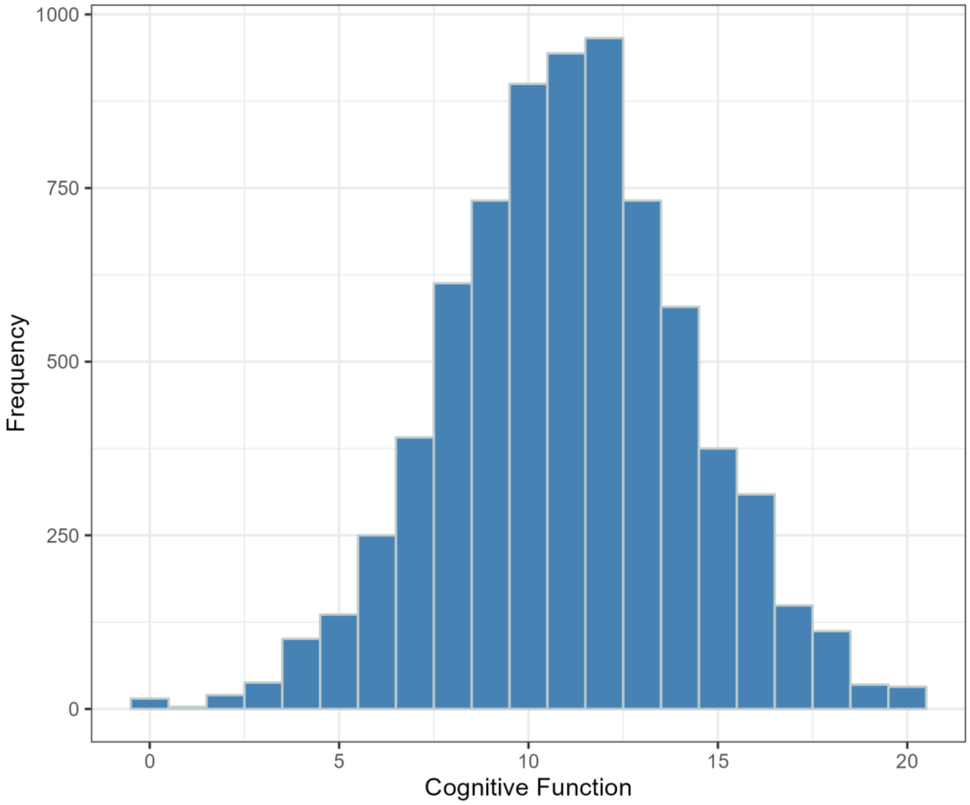

The x-axis is the total number of words recalled, while the y-axis is the number of participants.

Figure S5. Variable Importance

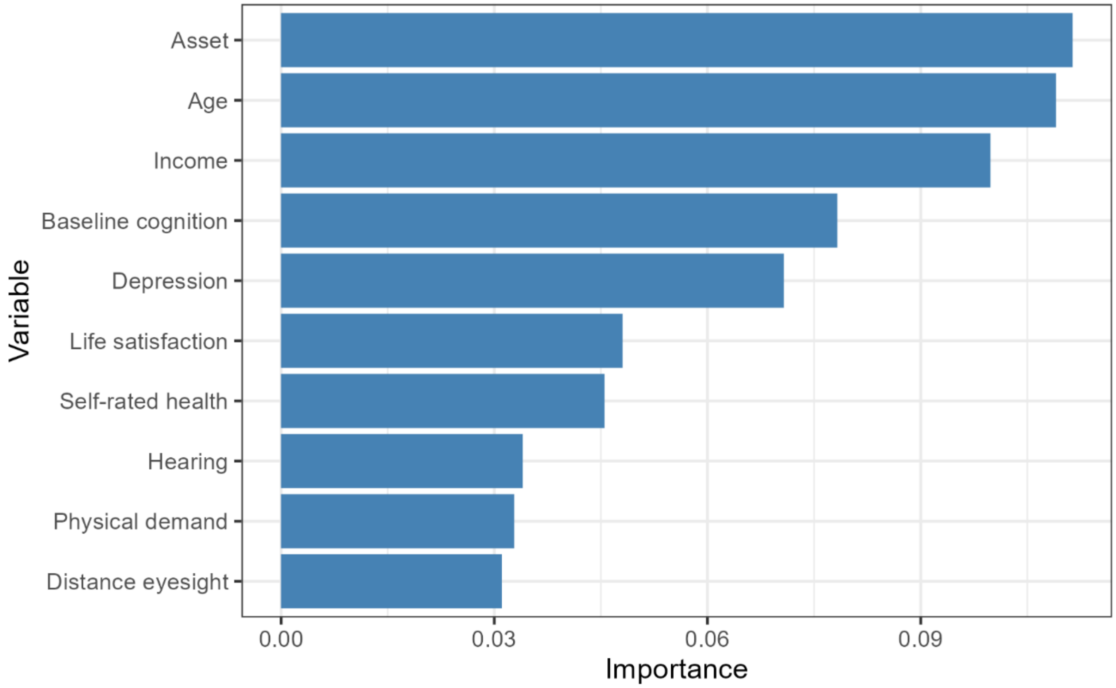

Figure S6. Distribution of Conditional Average Treatment Effects

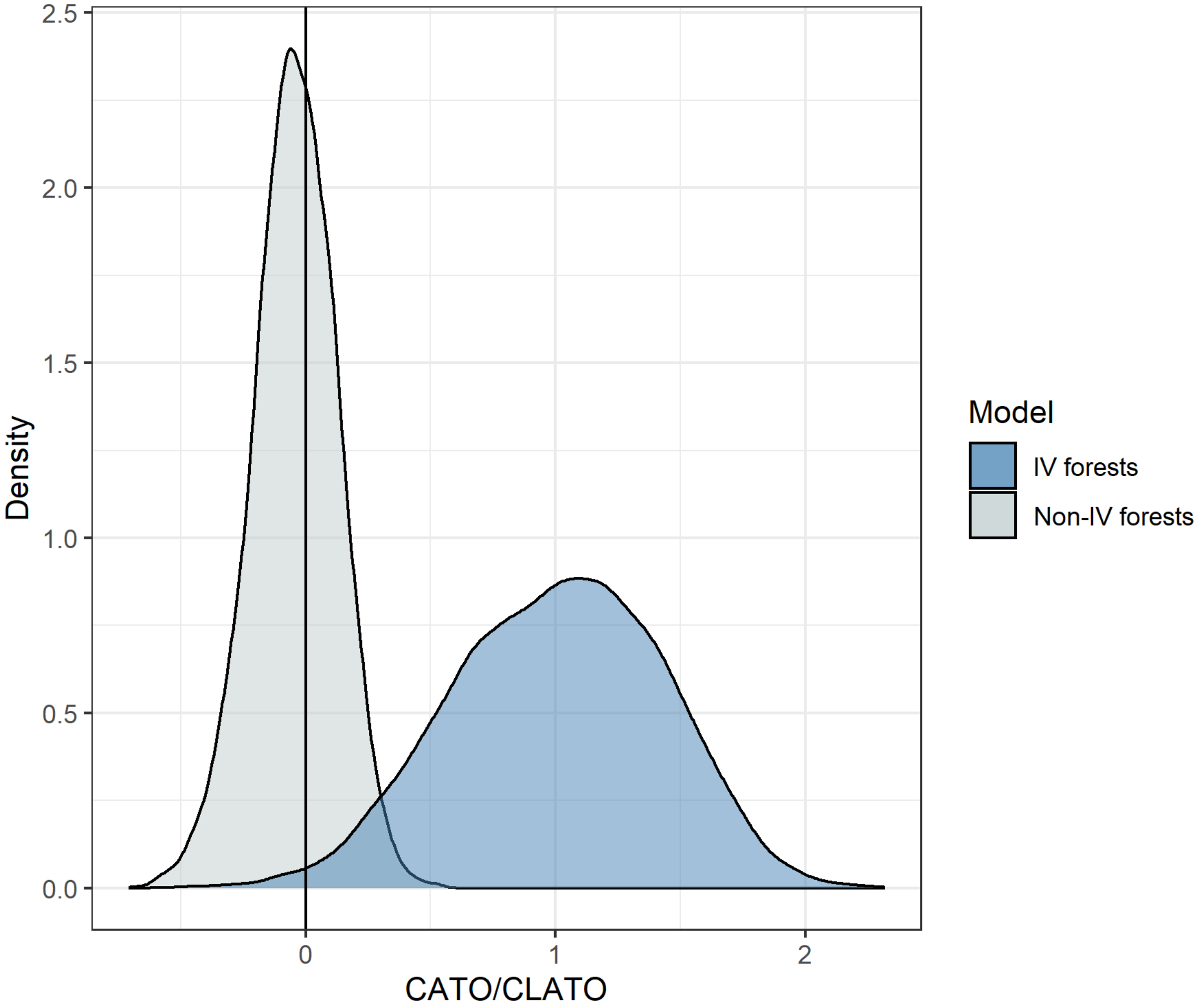

The estimate from non-IV forests represents the conditional average treatment effect on the overlap population (CATO), whereas the estimate from IV forests represents the conditional local average treatment effect on the overlap population (CLATO).

Figure S7. Calibration Plot for CLATO

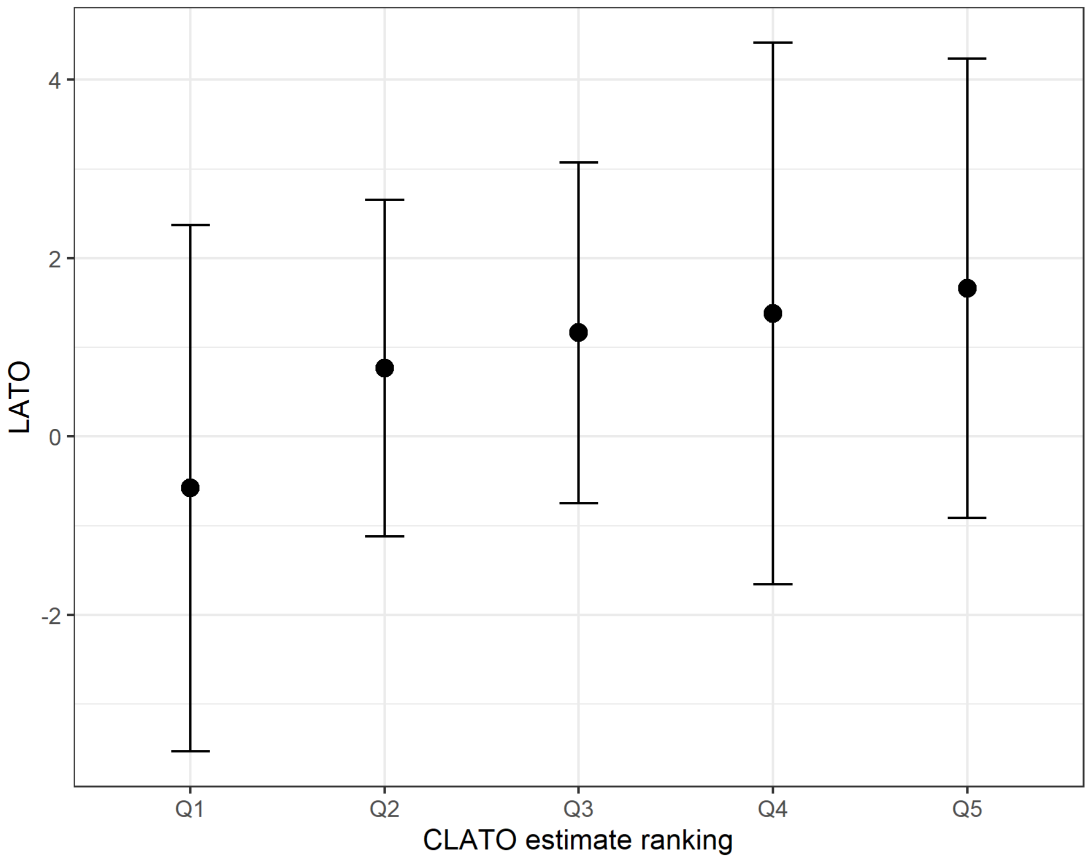

CLATO stands for the conditional local average treatment effect on the overlap population.

Figure S8. Partial Dependence Plot for Continuous Variables

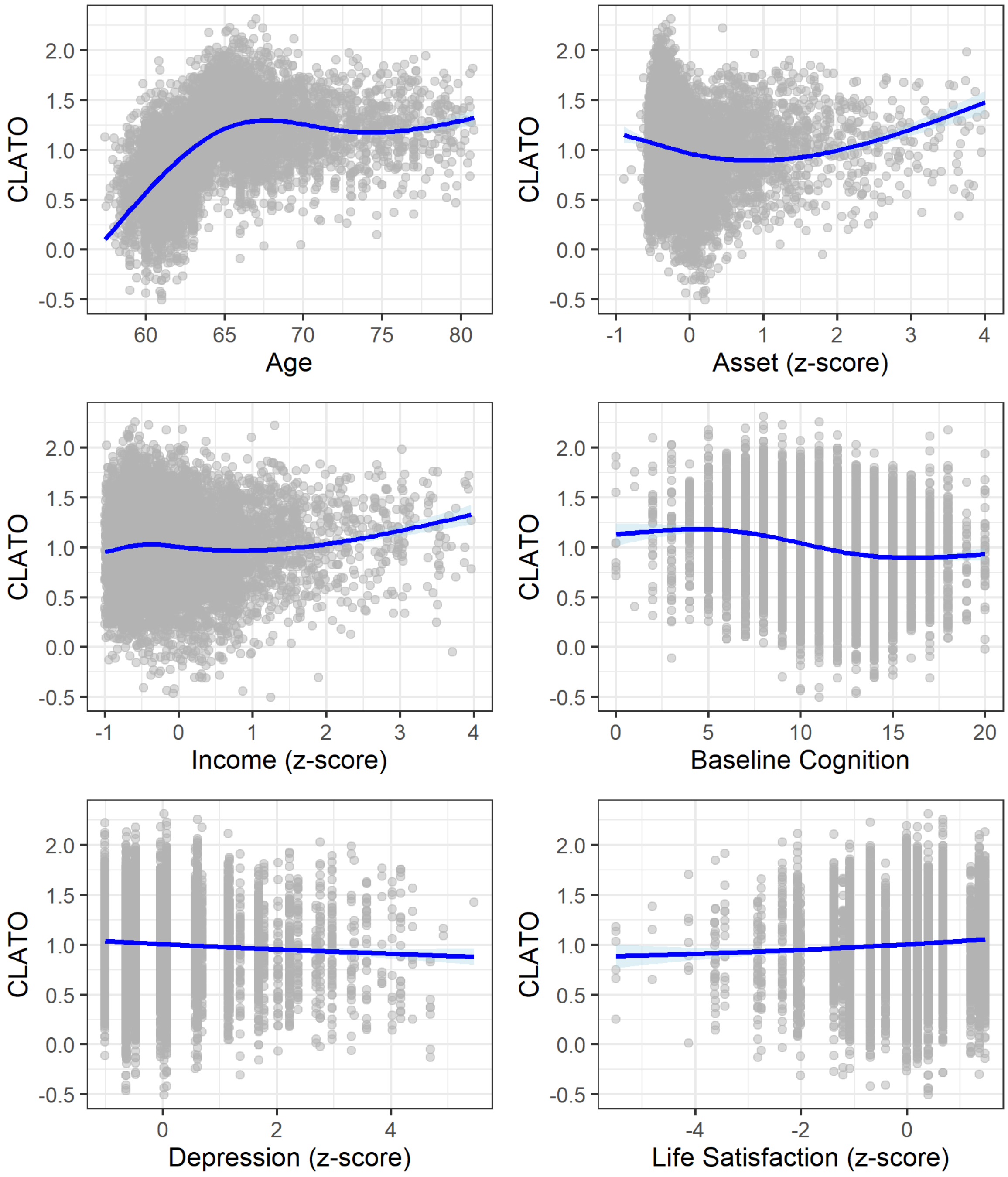

CLATO stands for the conditional local average treatment effect on the overlap population.

Table S7. Sensitivity Analyses

	Model 1	Model 2	Model 3	Model 4
Retirement	1.377	1.334	1.366	1.348
95% CI	(0.343 to 2.411)	(0.202 to 2.467)	(-0.040 to 2.773)	(0.174 to 2.523)
Observations	7268	6128	4582	5218

Model 1 restricted participants to individuals aged 55–75 years. Model 2 excluded individuals who mentioned retirement but worked in the second wave (i.e., partly retired), focusing solely on full retirement's impact on cognitive function. Model 3 excluded participants engaged in part-time jobs or were self-employed in the first wave, analyzing only full-time employees. Model 4 excluded data from the United States, the largest subset in our dataset. CI denotes confidence interval.

Supplementary References

1. Athey S, Tibshirani J, Wager S. Generalized random forests. *Ann Stat* 2019;**47**:1148–78.

2. Athey S, Imbens G. Recursive partitioning for heterogeneous causal effects. *Proc Natl Acad Sci U S A* 2016;**113**:7353–60.

3. Wager S, Athey S. Estimation and Inference of Heterogeneous Treatment Effects using Random Forests. *J Am Stat Assoc* 2018;**113**:1228–42.

4. Biewen M, Kugler P. Two-stage least squares random forests with an application to Angrist and Evans (1998). *Econ Lett* 2021;**204**:109893.

5. Chernozhukov V, Chetverikov D, Demirer M *et al.* Double/debiased machine learning for treatment and structural parameters. *Econom J* 2018;**21**:C1–68.

6. Crump RK, Hotz VJ, Imbens GW *et al.* Dealing with limited overlap in estimation of average treatment effects. *Biometrika* 2009;**96**:187–99.

7. Robinson P. Root- N-Consistent Semiparametric Regression. *Econometrica* 1988;**56**:931–54.

8. Angrisani M, Lee J. *Harmonization of Cross-National Studies of Aging to the Health and Retirement Study: Wealth Measures*. RAND Corporation, 2012.

9. Angrisani M, Lee J. *Harmonization of Cross-National Studies of Aging to the Health and Retirement Study: Income Measures*. RAND Corporation, 2012.

10. Hu P, Lee J. *Harmonization of Cross-National Studies of Aging to the Health and Retirement Study: Chronic Medical Conditions*. RAND Corporation, 2012.

11. World Health Organization. Obesity and overweight. *World Health Organ* 2021.

12. National Institute on Alcohol Abuse and Alcoholism. The Healthcare Professional's Core Resource on Alcohol. *Natl Inst Alcohol Abuse Alcohol* 2023.
